# Cryptic diversity in Zoraptera: *Latinozoros barberi* (Gurney, 1938) is a complex of at least three species (Zoraptera: Spiralizoridae)

**DOI:** 10.1371/journal.pone.0280113

**Published:** 2023-01-25

**Authors:** Petr Kočárek, Ivona Horká

**Affiliations:** Department of Biology and Ecology, University of Ostrava, Ostrava, Czech Republic; Universita degli Studi di Roma La Sapienza, ITALY

## Abstract

The order Zoraptera contains relatively few species, but current molecular phylogenetic studies suggest an unexpectedly high level of cryptic diversity in the order with many overlooked species based on morphology alone. *Latinozoros* Kukalova-Peck & Peck, 1993 represents the only genus of monotypic Latinozorinae (Zoraptera: Spiralizoridae) with only one species described, *L*. *barberi* (Gurney, 1938), until now. Although this species has been repeatedly reported from a number of locations in South and Central America, it is likely a complex of unrecognized species. Here, we present a molecular phylogenetic reconstruction revealing three genetically distinct lineages in *Latinozoros*, and we also present detailed morphological comparisons that prove the species status of *Latinozoros cacaoensis* sp. nov. from French Guiana and *L*. *gimmeli* sp. nov. from the Dominican Republic, Trinidad and Panama. The results indicate that the species previously referred to *L*. *barberi* is actually a species complex that includes *L*. *barberi*, the new species described here, and perhaps other species.

## Introduction

The order Zoraptera, which was described by Silvestri [1], is among the most enigmatic groups of insects. That the order was one of the last described in Insecta apparently stemmed not from the group’s rareness but rather from their cryptic life style and visual inconspicuousness. Although Zoraptera were discovered and established as an order more than 100 years ago, only 43 extant species have been described to date [2–4].

Zorapteran uniformity in general morphology led to the persistence of a conservative classification of extant Zoraptera, with only a single nominotypical genus in a single family for > 100 years [5]. The order was only recently classified based on phylogenetic analyses of molecular data [3].

Matsumura et al. [6] and Kočárek et al. [3] conducted molecular phylogenetic studies using a combination of nuclear and mitochondrial markers. Both of these independent analyses revealed two major phylogenetic lineages, which Kočárek et al. [3] classified as families (Zorotypidae Silvestri, 1913 and Spiralizoridae Kočárek, Horká & Kundrata, 2020), with each family divided into two robustly supported subclades, which were treated as subfamilies [3]. The recognition of two families and four subfamilies was supported by synapomorphies in the structure and shape of the male genitalia and in other taxonomically valuable characters including the number of spurs on the metatibia and the relative lengths of the first three antennomeres.

Both of the molecular phylogenetic studies [3,6] revealed several undescribed species that were found in all described families and subfamilies. This finding corresponds to the expected cryptic diversity, which has been pointed out by many previous authors [e.g., 2,5,7]. However, the morphological uniformity of the Zoraptera did not provide enough apomorphies for relationship assessment and for cladistic analyses, which can currently be substituted by molecular genetic information.

Here, we present descriptions of two new species of *Latinozoros* Kukalova-Peck & Peck, 1993 [28]; to date, the genus included only *L*. *barberi* (Gurney, 1938) [30]. We identified the additional species based on molecular phylogenetic analyses followed by rigorous morphological comparisons which led to the discovery of relevant diagnostic characters.

## Materials and methods

### Sampling and morphological study

An aspirator was used to collect zorapteran specimens from under the bark of different tree species; the specimens were stored in 96% ethanol. For observation of morphological and anatomical structures, specimens were placed in 10% KOH at room temperature for 1 h and was then washed with distilled water and returned to 96% ethanol for storage. Type specimens were slide-mounted in Euparal (BioQuip Products, Rancho Dominguez, California) or stored in 96% ethanol. The zorapteran specimens were studied and photographed with a Leica Z16 APO macroscope equipped with a Canon 6D Mark II camera; slide-mounted body parts and genitalia were observed and documented an Olympus CX41 microscope equipped with a Canon D1000 camera. Micrographs of 20 to 30 focal layers of the same specimen were combined with Helicon Focus software and finally processed with Adobe Photoshop CS6 Extended v13. For observation of genital armature, the armature was placed in 10% KOH at room temperature for 1 h before it was washed with distilled water and returned to 96% ethanol for observation and storage.

Zorapteran specimens used in this study were collected 1) during the expedition of the National Museum in Prague (Czech Republic) to the Dominican Republic in 2017 with the permission of the Ministerio de Medio Ambiente y Recursos Naturales República Dominicana, and 2) during expeditions to French Guiana by M. Kirstová (University of Ostrava, Czech Republic) in 2017 and by the author of this study (PK) in 2022. Because the material was collected outside of the protected areas, no permission was needed according to the regulations of French Guiana. All specimens have been deposited in the collection of the National Museum in Prague, Czech Republic.

Type depositories are abbreviated as follows: NMPC (National Museum, Prague, Czech Republic) and AMNH (American Museum of Natural History, New York, USA).

This study requires no ethics statement.

### Nomenclatural acts

The electronic edition of this article conforms to the requirements of the amended International Code of Zoological Nomenclature, and hence the new names contained herein are available under that Code from the electronic edition of this article. This published work and the nomenclatural acts it contains have been registered in ZooBank, the online registration system for the ICZN. The ZooBank LSIDs (Life Science Identifiers) can be resolved and the associated information viewed through any standard web browser by appending the LSID to the prefix "http://zoobank.org/". The LSID for this publication is: urn:lsid:zoobank.org:pub: FCB7178B-69BC-40F2-9823-2A8E3A3F4F5C. The electronic edition of this work was published in PLoS One with an ISSN 1932-6203.

### DNA analysis

Genomic DNA was extracted from tissues using the Qiamp DNA Micro Kit (Qiagen, Inc.) following the manufacturer’s protocols. Partial sequences of two nuclear (18S rRNA, histone 3) and one mitochondrial (16S rRNA) markers were amplified and sequenced. Polymerase chain reactions (PCR) were performed in 20-μl volumes containing 1 μl of DNA template, 0.4 μM of each primer, distilled water, and 1x PCRBIO HS Taq Mix Red (PCR Biosystems, London, UK). The primers and details of PCR conditions are indicated in S1 Table. The amplified DNA was purified using a Gel/PCR DNA Fragments Extraction Kit (GENAID, Taiwan). Sanger sequencing reactions were performed using an ABI3730XL DNA Sequencer by Macrogen (Amsterdam, The Netherlands).

The chromatograms were visually checked and manually edited where appropriate using ChromasPro v2.1.9 software (Technelysium, Brisbane, Australia). Details of analysed taxa including isolation numbers and GenBank accession numbers are indicated in Table 1. Classification and nomenclature follow Kočárek et al. [3]. Specimens published under the name *Latinozoros barberi* (Gurney, 1938) [30] without description or illustration of critical diagnostic characters (which would allow the assignment to one of the differentiated species) are presented as *Latinozoros* “*barberi*”.

**Table 1:** Details of the material used in the phylogenetic analyses. Newly obtained sequences are marked in bold. N/A–not available. For species in quotation marks, see Materials and Methods.

Analysed taxa	Sampling locality	Isolate	H3 (341 bp)	16S (∼554 bp)	18S (∼2,178 bp)	References
**Zorotypidae** Silvestri, 1913 [1]						
**Spermozorinae** Kočárek, Horká & Kundrata, 2020 [3]						
*Spermozoros asymmetricus* (Kočárek, 2017)	Brunei: Ulu Temburong	4Z	**ON811689**	MN790592	**ON807040**	[3]; this study
**Zorotypinae** Silvestri, 1913 [1]						
*Zorotypus delamarei* Paulian,1949	Madagascar	71Z	**ON811690**	MN790583	**ON807041**	[3]; this study
*Usazoros hubbadi* (Caudell,1918)	USA: Florida	89Z	**ON811691**	**ON722350**	**ON807042**	this study
**Spiralizoridae** Kočárek, Horká & Kundrata, 2020 [3]						
**Latinozorinae** Kočárek, Horká & Kundrata, 2020 [3]						
*Latinozoros barberi* (Gurney,1938) [30]	Brazil: Amazonas, Careiro	YK23	N/A	LC476757	LC477110	[6]
*Latinozoros cacaoensis* sp. nov.	French Guiana: Correze	48Z	**ON811693**	**ON722349**	**ON807044**	this study
*Latinozoros gimmeli* sp. nov.	Dominican Republic	41Z	**ON811692**	**ON722348**	**ON807043**	this study
**Spiralizorinae** Kočárek, Horká & Kundrata, 2020 [3]						
*Brazilozoros huxleyi* (Bolívar y Pieltain & Coronado, 1963)	Ecuador: Res. Biol. San Francisco	YK11	LC477129	LC476749	LC477105	[6]
*Brazilozoros weidneri* (New,1978)	Brazil: Amazonas	YK7	LC477124	LC476744	LC477101	[6]
*Centrozoros neotropicus* (Silvestri, 1916)	Costa Rica	43Z	N/A	MN790584	**ON807045**	[3]; this study
*Spiralizoros cervicornis* (Mashimo, Yoshizawa & Engel, 2013)	Brunei: Ulu Temburong	1Z	**ON811694**	MN790588	**ON807046**	[3]; this study
*Spiralizoros magnicaudelli* (Mashimo et al., 2013)	Malaysia: Sabah	6Z	**ON811695**	MN790600	**ON807047**	[3]; this study
**Outgroup (Polyneoptera)**						
Blattodea: *Gromphadorhina portentosa*	N/A	B01; N/A	AY125216	Z97626	Z97592	[8,9]
Dermaptera: *Euborellia arcanum*	USA: Florida	E8	**ON856150**	MN790595	MN790614	[3]; this study
Grylloblattodea: *Grylloblatta campodeiformis*	USA: MT, BuValo Horn Creek	BYU_gb31.1	DQ457398	DQ457262	DQ457299	[10]
Mantodea: *Mantis religiosa*	Ghana	MN247	FJ806794	FJ806243	FJ806432	[11]
Mantophasmatodea:*Tyrannophasma gladiator*	N/A; Namibia, Brandberg	BYU_ACMP002; BYU_mp02	AY521713	DQ457266	AY521863	[10,12]
Phasmatodea: *Heteropteryx dilatata*	West Malaysia (culture)	WS008	AY125241	KJ024429	AY121157	[9,13]
Pleoptera: *Malenka californica*	N/A	P01; BYU_PL001	AY338642	EF623182	AY338724	[14,15]

### Phylogenetic analyses

Sequences were aligned in MEGAX [16] using the MUSCLE algorithm [17], and the protein coding sequences (H3) were translated into amino acids to check for potential stop codons within the open reading frames. Substitution saturation was tested in DAMBE v6.4 [18] using the index proposed by Xia et al. [19]. Gblocks v0.91b was used to detect and eliminate poorly aligned and highly divergent regions in 16S and 18S rRNA alignments [20]. Genetic divergences between sequences were detected using the Kimura 2-parameter model within MEGAX software.

The multigene dataset was concatenated by SequenceMatrix v1.8 [21]. Bayesian inference (BI) and maximum likelihood (ML) analyses were used to estimate phylogenetic relationships, and both analyses were conducted with the on-line CIPRES Science Gateway v3.3 [22]. The best-fit partitioning schemes and molecular evolution models were selected under the corrected Akaike information criterion (AICc) using PartitionFinder v2.1.1 [23] (S2 Table).

Bayesian analysis was conducted with MrBayes v3.2.7a in XSEDE [24] using a Markov chain Monte Carlo (MCMC) method. Two independent MCMC runs of four chains were run 20 × 10^6^ generations (standard deviation of split frequencies <0.001). Trees were sampled every 100 generations, and 25% of the trees were discarded as burn-in. The convergence of BI analysis was confirmed in Tracer v1.6 [25].

The ML analysis using the GTR+G nucleotide model was conducted in RAxML-HPC BlackBox v8.2.12 [26]. The obtained trees were rooted by outgroup taxa from polyneopterous insect orders and were displayed using iTOL (interactive Tree Of Life) v6.5.6 [27].

### Map preparation

The distribution of *Latinozoros* species (Fig 10) has been projected onto a map obtained from *Natural Earth*, a freely available public domain under the Creative Commons Attribution License (CC BY 4.0) - https://www.naturalearthdata.com/.

## Results

### Taxonomy

Genus *Latinozoros* Kukalova-Peck & Peck, 1993

(syn. *Zorotypus* Silvestri, 1913, partim.)

urn:lsid:zoobank.org:act:7222D85F-D34F-4D41-8581-206E6809C3F7

Kukalova-Peck & Peck [28]: 342–343, 339 (description, illustration); Engel & Grimaldi [29]: 151–155 (synonymy with *Zorotypus* Silvestri, 1913) [1]; Kočárek, Horká & Kundrata [3]: 11–13 (reinstated as valid genus, diagnosis, illustration).

Type species. *Zorotypus barberi* Gurney, 1938 [30]; designated by Kukalova-Peck & Peck [28]: 342–343.

### Diagnosis

Body length 1.9–3.1 mm, basic color of apterons from ochre to light brown, alates and dealates darker. Antenna with nine antennomeres, antennomeres I (scaphus) and II (pedicellus) slightly curved, antennomere I long, as long as antennomeres II+III combined, antennomere II short, slightly shorter than antennomere III; antennomeres IV–IX short, approximately 1.5–2.0 times longer than wide, distally narrowed. Small cephalic gland present in the centre of vertex. Pronotum subrectangular, slightly wider than long. Forewing with pterostigma long, inclined to extend posteriorly over weak posterior boundary. C—R + RA area narrow, RA almost straight, Rp-mp brace varies from a cross-vein to a short fusion. MP + CuA distinctly shorter than the following proximal portion of MP, both forming a straight line, CuA3 + 4 completely lost. Posterior margin between CuA3 + 4 and apical margin almost straight. Metafemur swollen basally, ventral surface with 7–9 spurs; metatibia with two stout spurs, one of which is located apically; paired claws hooked, with dilated empodium, shorter than one-third length of claw.

Male abdominal tergite T10 separated into anterior and posterior parts with pair of groups of thinner setae arranged as short comb (ctenidium) on both sides. Male abdominal tergite T10 with spatula-like projection, tergite T11 with hooked median projection. Cerci unsegmented, conical, two times longer than wide. Male genitalia symmetrical, composed of a pair of dorsal lobate sclerites (sclerite Ia,b), mesal sclerite (sclerite II), a pair of ventral sclerites (sclerite IIIa,b), and weakly sclerotized (membranous) basal plate with tongue-like anterior projection with rod-like paired sclerites medially (sclerites IVa,b). Intromittent organ long, encircling anterior projection of basal plate.

Taxa included: *Latinozoros barberi* (Gurney, 1938), *Latinozoros cacaoensis* sp. nov., *Latinozoros gimmeli* sp. nov.

Distribution: Costa Rica: Cocos Island [30]; Costa Rica [31]; Panama [31,32]; Venezuela [28,33], French Guiana [33]; Dominican Republic [31]; Trinidad [34]; Brazil [6]; Puerto Rico [35].

**Fig 1:**
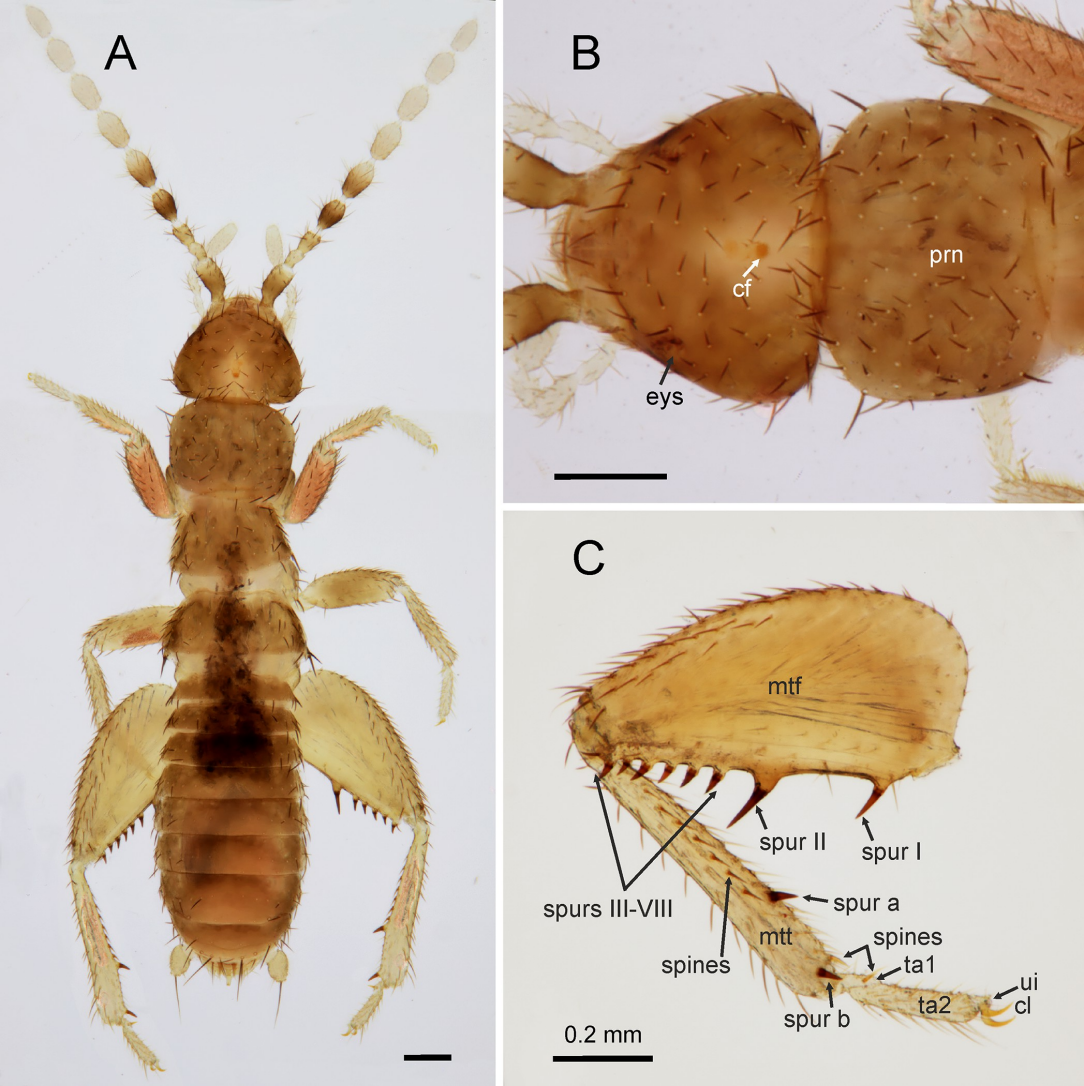
*Latinozoros cacaoensis* sp. nov., apterous male paratype. A: Dorsal view; B: Dorsal view of head and pronotum; C: Left metaleg, anterior view. Abbreviations: cl–clavus; ui–unguitractor plate; eys–eye spot; cf–cephalic gland; prn–pronotum; mtf–metafemur; mtt–metatibia; ta–tarsus. Scale bars = 0.2 mm.

**Fig 2:**
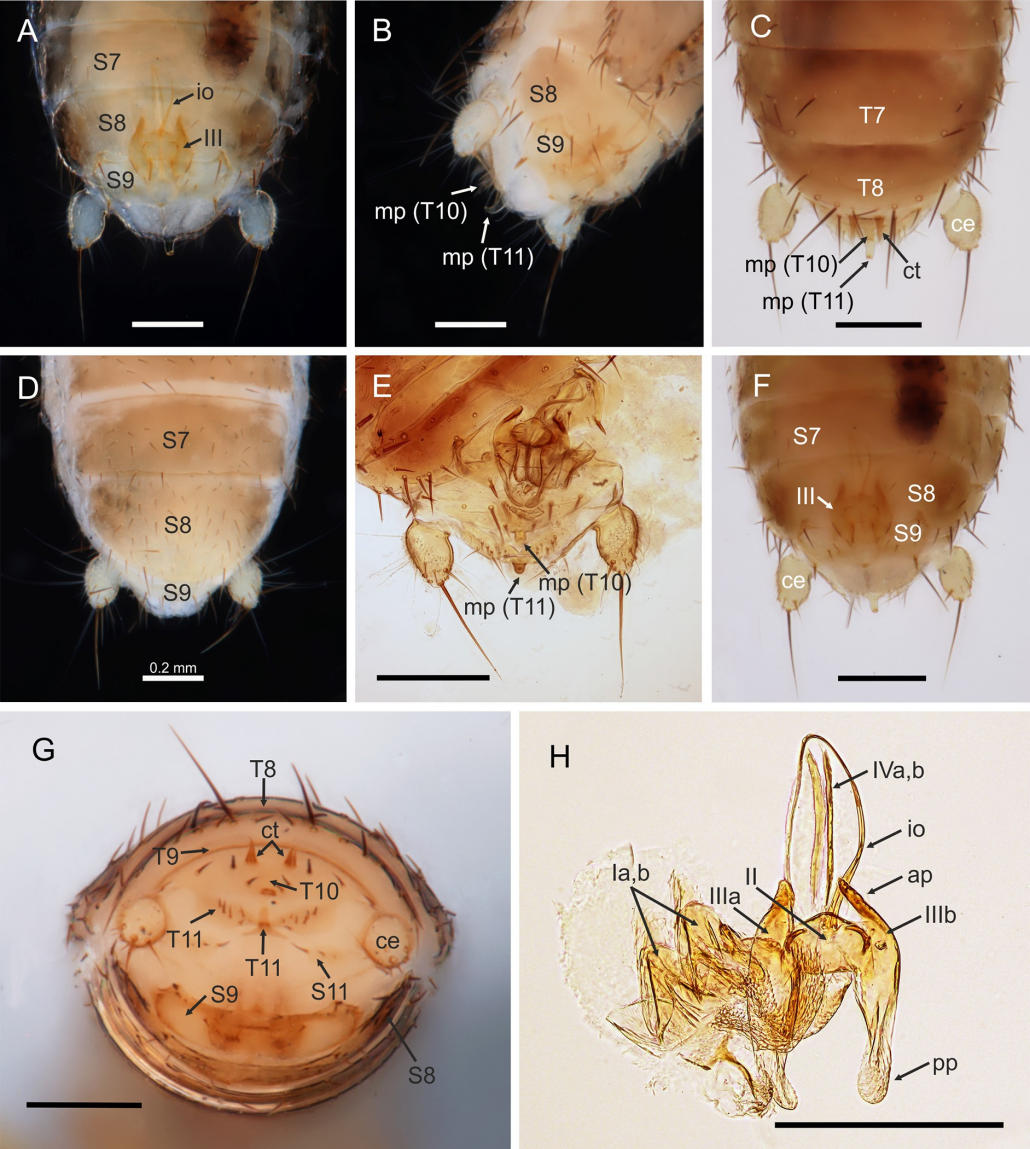
*Latinozoros cacaoensis* sp. nov., apterous male and female paratypes. A: Posterior segments of male abdomen, ventral view; B: Posterior segments of male abdomen, latero-ventral view; C: Posterior segments of male abdomen, dorsal view; D: Posterior segments of female abdomen, ventral view; E: Posterior segments of male abdomen with partly dissected genital, ventral view; F: Posterior segments of male abdomen, ventral view; G: Tip of male abdomen, posterior view; H: Dissected male genital, ventral view. Abbreviations: ap–anterior process of ventral sclerite (III) of male genital; ce–cercus; ct–ctenidium; io–intromittent organ of male genital; mp–median up-curved projection; pp–posterior process of ventral sclerite (III) of male genital; S–abdominal sternite; T–abdominal tergite; I–dorsal sclerites of male genital; II–mesal sclerite of male genital; III–ventral sclerites of male genital; IV—rod-like paired sclerites of male genital. Scale bars = 0.2 mm.

**Fig 3:**
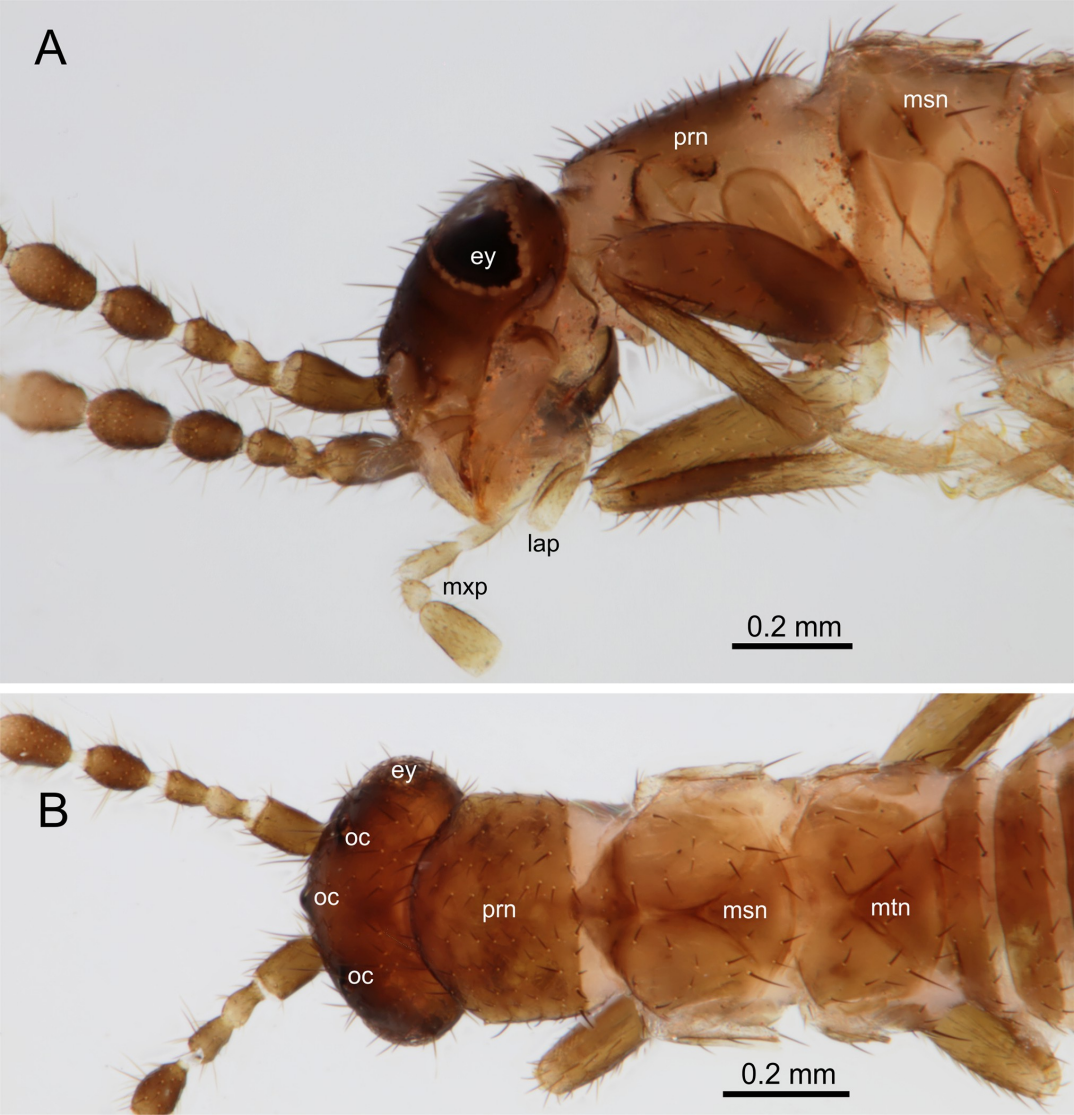
*Latinozoros cacaoensis* sp. nov., dealate female paratype. A: Ventro-lateral view of head and thorax; B: Dorsal view of head and thorax. Abbreviations: ey–compound eye; oc–ocelli; lap–labial palpus; prn–pronotum; msn–metanotum; mtn–metanotum; mxp–maxillary palpus. Scale bars = 0.2 mm.

*Latinozoros cacaoensis* sp. nov.

urn:lsid:zoobank.org:act:44F9FB22-AAEB-4D6B-A3D5-F11C04736640; Figs 1–3.

Type locality. French Guiana: Cacao.

Material examined. Holotype male, labelled ’French Guiana, Cacao env., Molokoï track, 4°33’39.70"N, 52°27’44.52"W, 40 m, 10.6.2022, P. Kočárek, M. Jankásek, I.H. Tuf leg.’ (NMPC); paratypes with same data as holotype: 1 male,1 female (NMPC); 1 male,1 female paratypes labelled: ’French Guiana, Montsinéry env., Sentier du Bagne des Annamites track, 4°49’37.23"N, 52°30’58.24"W, 25 m, 11.6.2022, P. Kočárek, M. Jankásek, I.H. Tuf leg.’ (NMPC); 1 male,1 female paratypes labelled: ’ S America: French Guiana, Correze env., 4°30’52"N, 52°20’13"W, 26 m, 25.1.2016, M. Kirstová leg. (NMPC).

### Diagnosis

*Latinozoros cacaoensis* sp. nov. (Figs 1–3) is morphologically similar to both *L*. *barberi* (Gurney, 1938) and *L*. *gimmeli* sp. nov., but it can be distinguished by the specific shape of the male genitals. The anterior process of the basal plate is short in *L*. *cacaoensis* sp. nov., as long as the ventral sclerites (sclerite III); the posterior processes of the ventral sclerites are 2 times longer than anterior processes of the ventral sclerites, posterior processes with broadly rounded apex (Fig 2H). In *L*. *barberi*, the anterior process of the basal plate is 3 times longer than the ventral sclerites, at least 3 times longer than the ventral sclerites, and the distal processes of ventral sclerites are very broad and rounded. In *L*. *gimmeli* sp. nov. the anterior process of the basal plate is shorter, about 1.5 times longer than the ventral sclerites; the distal processes of the ventral sclerite are narrowed to needle-like process. The species also differs in the arrangement of spurs on the metafemur; the ventral surface of the metafemur bears 7–8 stout long spurs in *L*. *cacaoensis* sp. nov. (Fig 1C) but 9 stout spurs in *L*. *barberi* and *L*. *gimmeli* sp. nov. Females of *L*. *cacaoensis* sp. nov. and *L*. *gimmeli* sp. nov. differ also in the shape of the eight sternite (S8). The distal end of S8 is broadly rounded in *L*. *cacaoensis* sp. nov. (Fig 2D), but the middle part of the distal end of S8 extends to a triangular projection in *L*. *gimmeli* sp. nov. The shape of S8 in female of *L*. *barberi* is unknown, because females of this species have not been described.

### Description of apterous male

Total body length 2.86–3.11 mm, head width 0.56–0.57 mm, antenna length 1.46–1.48 mm, pronotal width 0.54–0.55 mm, metafemur length 0.86–0.88 mm, metatibia length 0.76–0.77, abdomen maximal width 0.64–0.66 mm, cerci length 0.15 mm. Body color pale brown, legs, cerci and membranous regions lighter, antennae light with antennomeres I and III-V partly darkened (Fig 1A). Head subtriangular, slightly wider than pronotum (Fig 1A and 1B); cephalic setae (Fig 1B) short and sparse, not grouped; compound eyes and ocelli absent, vestigial eyespots visible; cephalic gland present in centre of the head, with several short setae; antennae 9-segmented (Fig 1A), antennomere I (scaphus) slightly curved outward, antennomere II (pedicellus) slightly curved, short, about one-third length of antennomere I; antennomere III slightly longer than antennomere II, antennomeres IV–IX longer than wide, distally narrowed. Mandibles asymmetrical, each mandible with four apical teeth and well-developed molar region; maxillary palpus five-segmented, labial palpus three-segmented. Pronotum subrectangular, only slightly wider than long, slightly narrowed posteriorly and setose, chaetotaxy as depicted in Fig 1A and 1B; mesonotum trapezoidal, shorter than pronotum; metanotum trapezoidal, distinctly wider than long, shorter than mesonotum. Legs with short setae (Fig 1A and 1C); posterior surface of profemur covered with longer setae; protibia with apical spur; mesofemur slightly narrower than profemur, dorsal surface covered with longer setae than ventral part; mesotibia covered with short setae and two apical spurs; metafemur broad, expanded, gradually tapering toward apex (Fig 1C), dorsal surface densely setose, middle part posteriorly without setae, ventral surface with 7‒8 stout spurs situated on tubercles, slightly angled toward metafemoral apex; proximal spur I thinner than spur II, length about ¾ of spur II; second spur (spur II) long and stout; spurs III‒VII(VIII) short, length is 2/3 of spur II, spurs III-VII(VIII) close to each other (Fig 1C); metatibia with short setae and two strongly sclerotized spurs ventrally (spur a, b), length similar to length of metafemoral spurs III-VIII, one situated in basal third of metatibia (spur a), second on apex posteriorly (spur b) together with prominent, but not strongly sclerotized spine; ventral part of metatibia with row of prominent, moderately strong spines; basitarsus (tarsomere I) with prominent spine in distal third on ventral side. Distal end of tarsomere II with unguitractor plate and hooked paired claws (Fig 1C).

Abdominal tergites I-III (T1-T3) with stronger setae on posterior parts of lateral margins, middle part without setation; abdominal tergites IV-VIII (T1-T8) with sparse setation along lateral margins, dorsal surface without setae, distal edge of T8 lined by several long setae, T9 short, weakly sclerotized (Fig 2C); T10 weakly sclerotized, separated into anterior and posterior parts, anterior part with three short setae along anterior margin and two groups of thinner setae arranged as short comb (ctenidium) on both sides, posterior half mostly membranous, central region with median wrench-like, slightly upcurved projection (Fig 2A–2C and 2E–2G), T11 weakly sclerotized, bearing moderately long setae (for chaetotaxy, see Fig 2C, 2E and 2G) with long, evenly upcurved median projection, with its apex above level of projection of T10, epiproct and paraproct unsclerotized; cerci (Fig 2A–2C and 2E–2G) unsegmented, longer than wide, conical with slightly pointed apex, covered with numerous minute spicules and shortly setose, with one long distally oriented seta.

Abdominal sternite I (S1) scarcely sclerotized; sternites S2-S7 with sparse setation along lateral margins, middle part without setation, S8 with weak lateral depressions distally, each depression with one longer seta, middle part of distal edge (between depressions) nearly flat, S9 narrow, distal corners with longer setae, middle part of sternite with several longer setae, midpart of the distal edge pointed, S10 invisible externally, beneath S9, S11 with two lateral hemitergites, each with several 2–3 setae of short and moderate length (Fig 2A–2C and 2E–2G).

Male genitalia symmetrical (Fig 2H), composed of pair of dorsal lobate sclerites (sclerite Ia,b), mesal sclerite with convex anterior margin and flat posterior margin (sclerite II), and pair of ventral sclerites (sclerite IIIa,b) with pointed anterior processes and broadly rounded posterior processes covered by weak spines; inner parts of ventral sclerites with small sclerotized needle-like curved processes oriented antero-ventrally; weakly sclerotized (membranous) basal plate with tongue-like anterior process with rod-like paired sclerites medially (sclerites IVa,b). Intromittent organ long, encircling anterior process of basal plate (Fig 2H). Anterior process of basal plate short, as long as ventral sclerite.

### Description of apterous female

Similar to male except the following features: head without visible cephalic gland; metafemur slender, ventral surface with same arrangement of spines as in males, but spines thinner; abdomen wider, maximal width 0.77–0.71–0.74 mm, abdominal T8 uniformly sclerotized with 6‒8 short setae on each side and pair of longer setae; S8 strongly trapezoidal, wider than long, with short setae evenly scattered and longer setae flanking the distal and lateral edges; distal end regularly rounded, without projection (Fig 2D); S9 short and trapezoidal with several small setae along posterior margin.

### Description of dealate female

The features of dealate female generally similar to those of the apterous female except as follows: blackish brown coloration. Compound eyes and three black ocelli present. Distal quarter of pronotum only weakly sclerotized, mesonotum and metanotum indistinctly divided into slightly pointed prescutum, large scutum, and smaller posteriorly rounded scutellum (Fig 3A and 3B).

### Molecular barcode

We obtained partial 16S rRNA sequence (509 bp) of *L*. *cacaoensis* sp. nov. as DNA barcode for the purpose of molecular identification of the species, and we deposited it in GenBank under accession number ON722349.

### Etymology

The species is named according to the type locality, the village Cacao in French Guiana.

### Distribution, habitat, and biology

**Fig 4:**
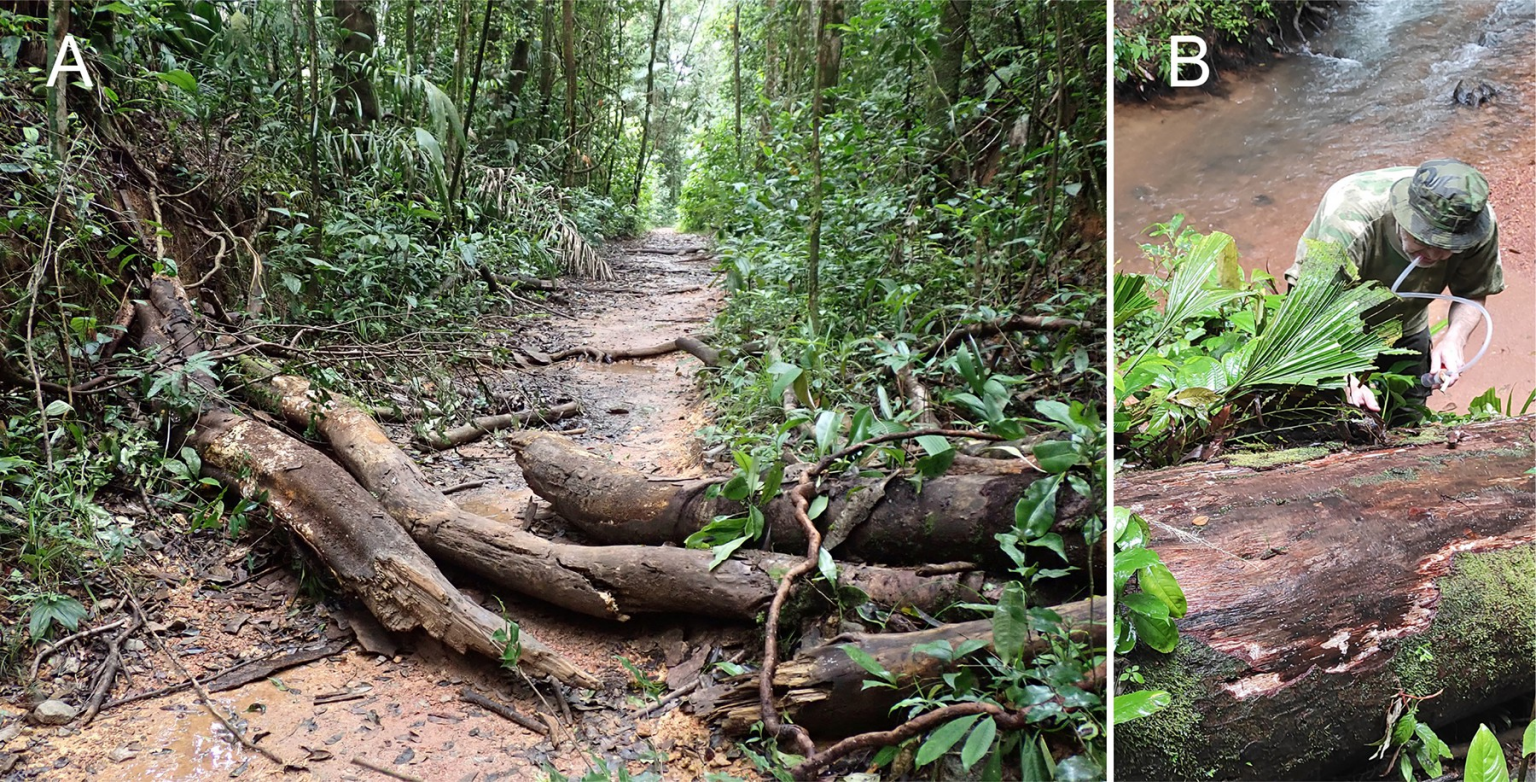
Habitat of *Latinozoros cacaoensis* sp. nov. A: Rotting logs on Sentier du Bagne des Annamites track (French Guiana: Montsinéry env.) where paratypes were collected. Photo: P. Kočárek; B: collecting of *L*. *cacaoensis* sp. nov. by the author (PK) at the type locality on Molokoï track (French Guiana: Cacao env.). Photo: I.H. Tuf.

**Fig 5:**
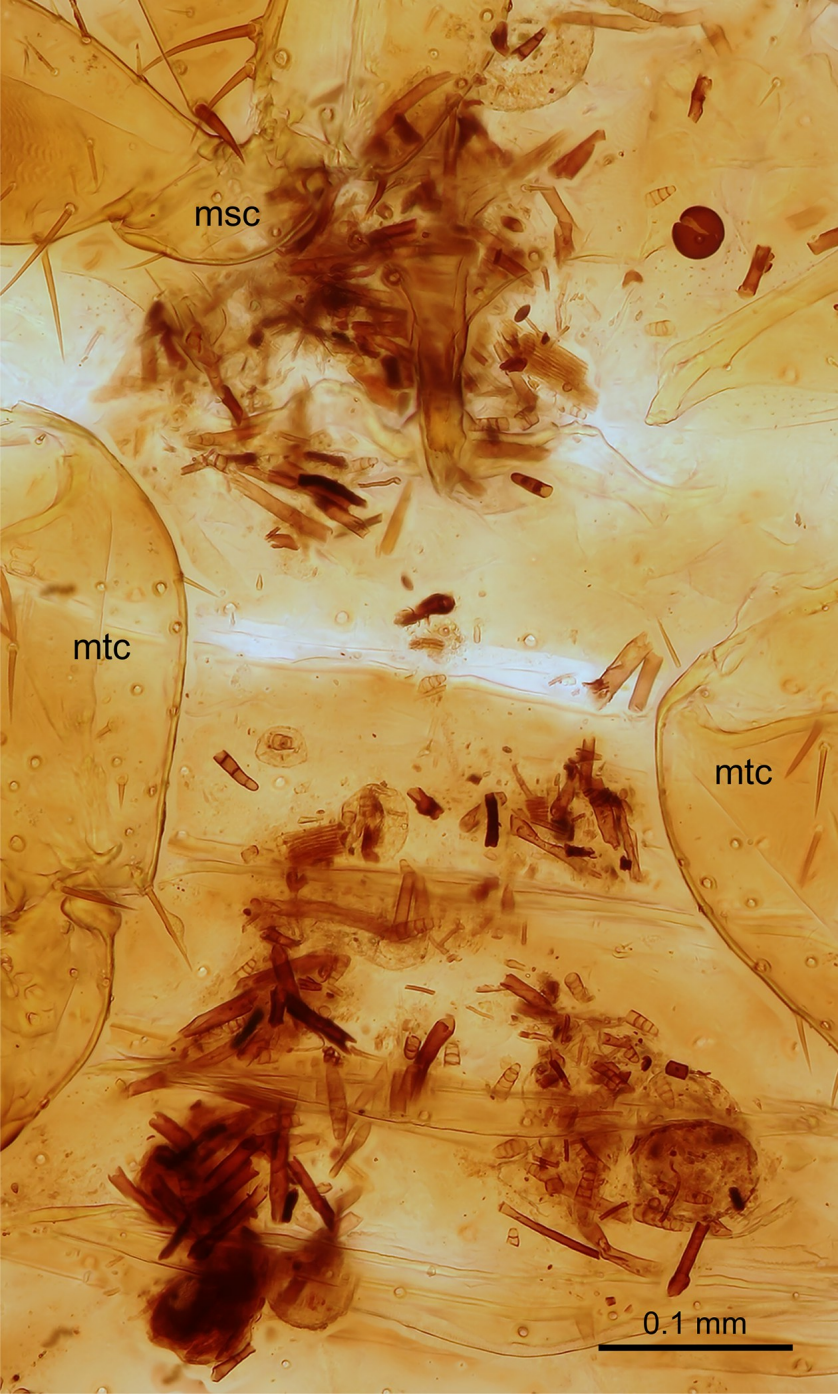
*Latinozoros cacaoensis* sp. nov., gut content inside male paratype abdomen, ventral view. Abbreviations: msc–mesocoxa; mtc–metacoxa. Scale bar = 0.1 mm.

*Latinozoros cacaoensis* sp. nov. was collected under the bark of rotting logs in lowland rainforest (Fig 4A and 4B). The translucent abdomen of samples, lightened by KOH solution, enabled observation and partial identification of gastrointestinal tract content (Fig 5), which was composed of fungal hyphae, spores, and fragments of fibre sclerenchyma tissue. Chyme composition points to detritovory with preference for fungi. The species is currently known only from French Guiana, but we expect its occurrence in similar habitats in neighbouring countries in Amazonia.

**Fig 6:**
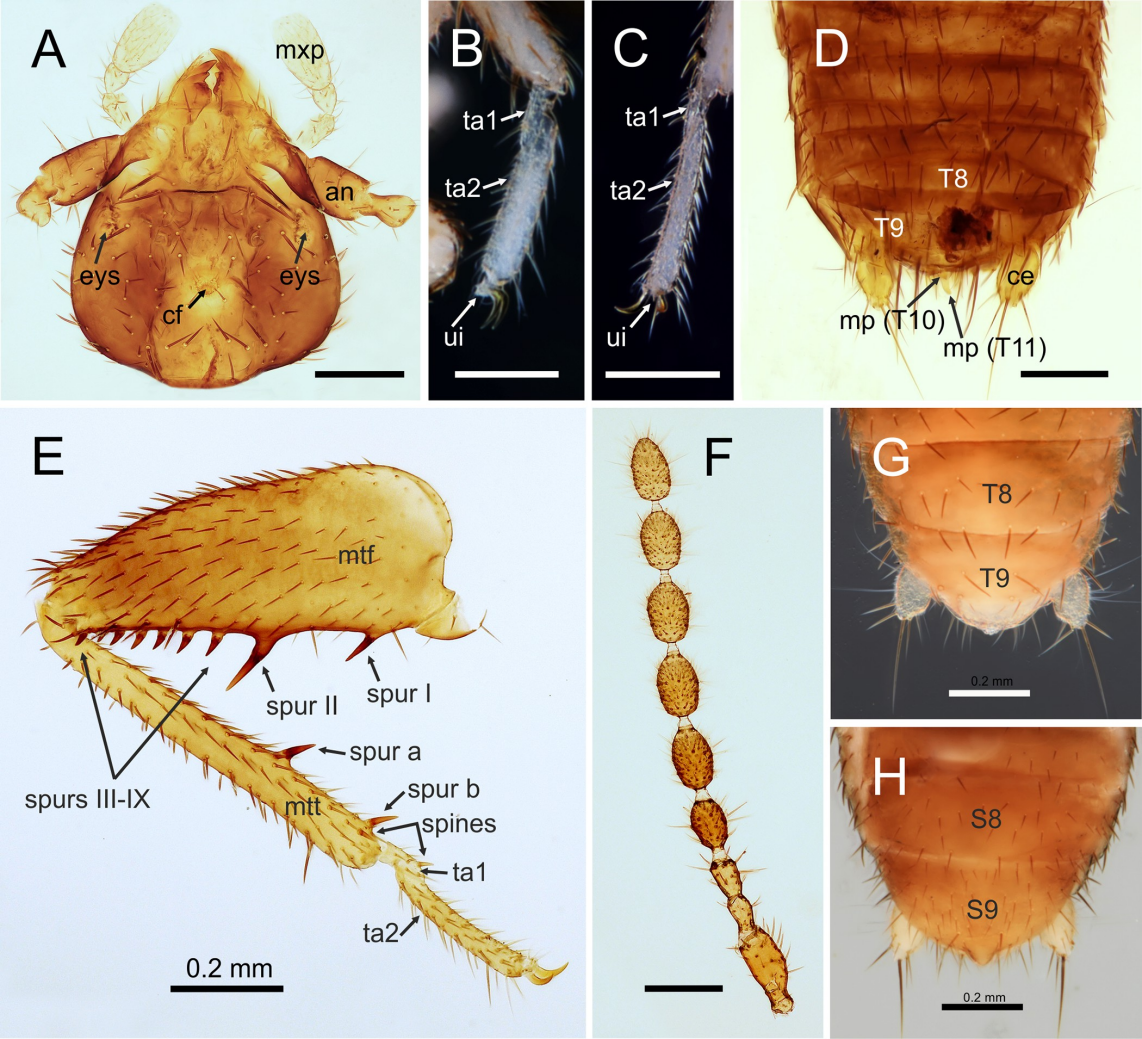
*Latinozoros gimmeli* sp. nov., apterous male and female paratypes. A: Head of male, dorsal view; B: Tarsi of right foreleg, female; C: Tarsi of left hind leg, female; D: Posterior segments of male abdomen, dorsal view; E: Right metaleg of male, lateral view.; F: Left antenna of male, dorsal view; G: Posterior segments of female abdomen, dorsal view; H: Posterior segments of female abdomen, ventral view. Abbreviations: an–antenna; eys–eye spot; ce–cercus; cf–cephalic gland; ct–ctenidium; mp–median up-curved projection; mtf–metafemur; mtt–metatibia; mxp–maxillary palp; ta–tarsus; ui–unguitractor plate; S–abdominal sternite; T–abdominal tergite. Scale bars = 0.2 mm.

**Fig 7:**
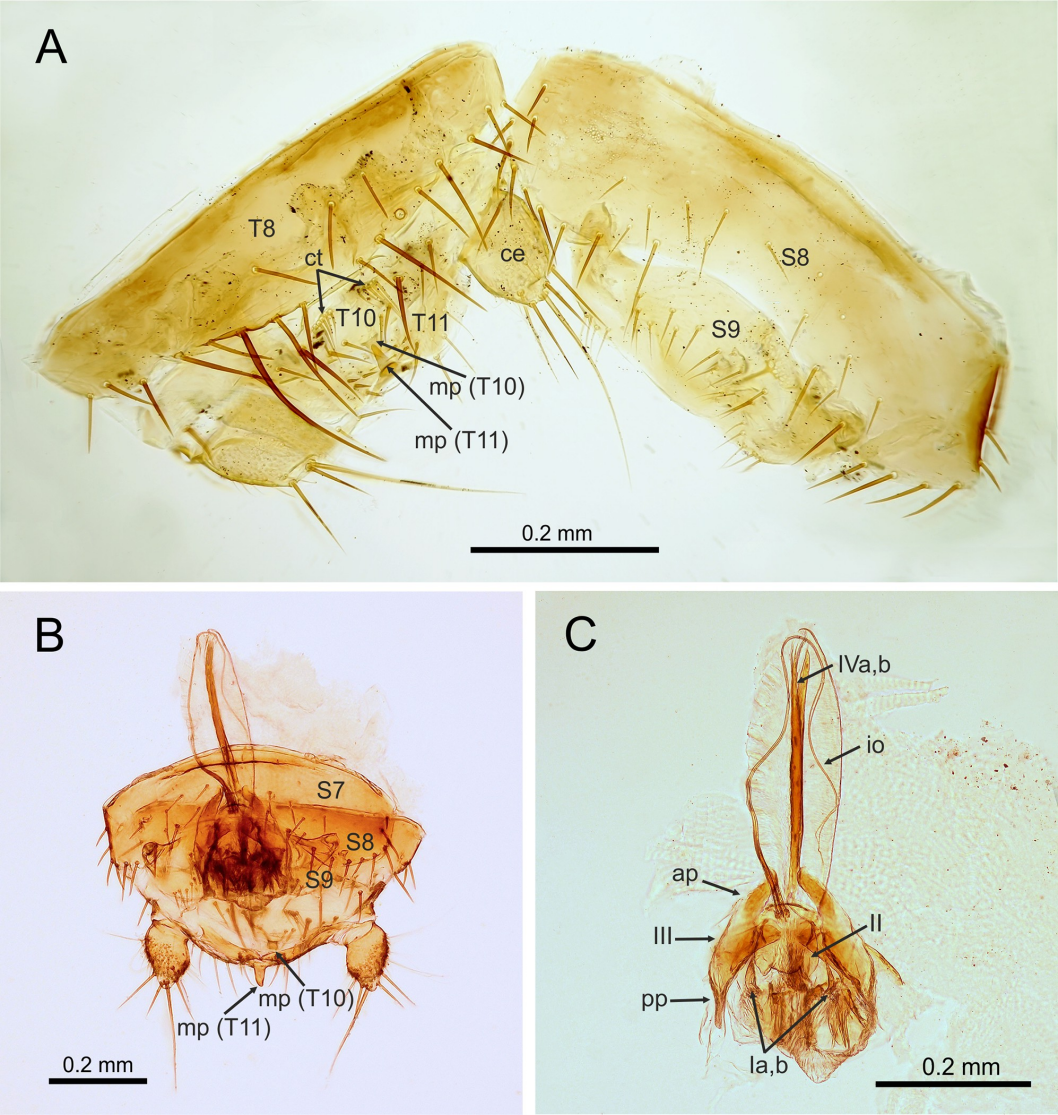
*Latinozoros gimmeli* sp. nov., apterous male paratypes. A: Posterior segments of male abdomen, dissected; B: Posterior segments of male abdomen, partly dissected, ventral view; C: Dissected male genital, ventral view. Abbreviations: ap–anterior process of ventral sclerite (III) of male genital; ce–cercus; ct–ctenidium; io–intromittent organ of male genital; mp–median up-curved projection; pp–posterior process of ventral sclerite (III) of male genital; S–abdominal sternite; T–abdominal tergite; I–dorsal sclerites of male genital; II–mesal sclerite of male genital; III–ventral sclerites of male genital; IV—rod-like paired sclerites of male genital. Scale bars = 0.2 mm.

*Latinozoros gimmeli* sp. nov.

urn:lsid:zoobank.org:act:C0C9487A-E595-4EAE-92A2-081C9AE8350E; Figs 6 and 7.

Type locality. Dominican Republic: Baharona Prov.

Material examined. Holotype male (dissected), labelled ’Dominican Rep.: Barahona, MN Domingo Fuerte “Cachote”, 18°4.48´N 71°11.03´W, 1188 m, 14.viii.2014, Deler, Fikáček, Gimmel DR04 // under bark of wet, rotten *Pinus occidentalis*’ (NMPC); paratypes with same data as for holotype: 2 females (NMPC); additional material examined: ’Trinidad: Northern range, Las lapas W. of summit of Blanchisseuse Road, 1800 ft, 18. February 1964, J. G. Rozen & P. Wygodzinsky’, 1 female (AMNH: IZC00321203).

### Diagnosis

*Latinozoros gimmeli* sp. nov. (Figs 6 and 7) is morphologically similar to both *L*. *barberi* (Gurney, 1938) [30] and *L*. *cacaoensis* sp. nov., and can be distinguished by the specific shape of its male genitals. The anterior process of the basal plate of the male genital is about 1.5 times longer than the ventral sclerites in *L*. *gimmeli* sp. nov., and the distal processes of the ventral sclerites are long and narrowed (Fig 7B and 7C). In *L*. *barberi*, the anterior process of the basal plate is long, at least 3 times longer than the ventral sclerites, and the distal processes of the ventral sclerite are very broad and rounded; in *L*. *cacaoensis* sp. nov. the anterior process of the basal plate is short, as long as the ventral sclerite (sclerite III), the posterior processes of the ventral sclerites are 2 times longer than anterior processes, with broadly rounded apex. Difference is also in the arrangement of the spurs on metafemur, whereas the ventral surface of metafemur bears 9 stout spurs in *L*. *barberi* and *L*. *gimmeli* sp. nov. (Fig 6E) but only 7–8 stout spurs in *L*. *cacaoensis* sp. nov. (Fig 1C). Females of *L*. *cacaoensis* sp. nov. and *L*. *gimmeli* sp. nov. differ also in the shape of the eight sternite (S8). Middle part of the distal end of S8 extends to triangular projection in *L*. *gimmeli* sp. nov. (Fig 6H), but the distal end of S8 is broadly rounded in *L*. *cacaoensis* sp. nov. (Fig 2D). Shape of S8 in female of *L*. *barberi* is unknown, because the female of this species is not described.

### Description of apterous male

Total body length 2.78 mm, head width 0.62 mm, antenna length 1.58 mm, pronotal width 0.57 mm, metafemur length 0.82 mm, metatibia length 0.84, abdomen maximal width 0.71 mm, cerci length 0.16 mm. Body color pale brown; legs, cerci and membranous regions lighter. Head subtriangular, longer than wide (Fig 6A), slightly wider than pronotum, distal edge convex medially; cephalic setae (Fig 6A) short and sparse, not grouped; compound eyes and ocelli absent, vestigal eyespots visible; cephalic gland present in centre of head, with several short setae; antennae 9-segmented (Fig 6F); antennomere I slightly curved outward, as well as antennomere II, antennomere II short, about one-third length of antennomere I; antennomere III slightly longer than antennomere II, length of antennomeres II+III similar to length of antennomere I, antennomeres III–IX longer than wide, distally narrowed. Mandibl asymmetrical, each mandible with four apical teeth and well-developed molar region; maxillary palpus five-segmented, labial palpus three-segmented. Pronotum subrectangular, slightly wider than long, slightly narrowed posteriorly and setose; mesonotum trapezoidal, shorter than pronotum; metanotum trapezoidal, distinctly wider than long, shorter than mesonotum. Legs with short setae; posterior surface of profemur covered with longer setae; protibia with apical spur; mesofemur slightly narrower than profemur, dorsal surface covered with longer setae than ventral part; mesotibia covered with short setae and two apical spurs; metafemur broad, expanded, irregularly tapering to apex (Fig 6A), anterior surface broadly setose, posterior part sparsely setae, ventral surface with 9 stout spurs situated on tubercles, slightly angled toward metafemoral apex, proximal spur I thinner and shorter than spur II, length about 2/3 of spur II, second spur (spur II) long and stout, spurs III‒IX short, length about half of spur II, spurs III-IX close to each other (Fig 6A); metatibia with short setae and two strongly sclerotized spurs ventrally (spur a, b), length similar to length of metafemoral spur I, one situated in basal third of metatibia (spur a), second in apex posteriorly (spur b) together with prominent, but not strongly sclerotized spine; basitarsus (tarsomere I) with prominent spine in distal third ventrally. Distal end of tarsomere II with dilated unguitractor plate, paired claws hooked (Fig 6B and 6C).

Abdominal tergites I-III (T1-T3) with longer setae in posterior parts of lateral margins, middle part without sparse setation, abdominal tergites IV-VIII (T1-T8) with regular sparse setation, distal edge of T8 lined by several long setae (Figs 6B and 7A), T9 short, weakly sclerotized; T10 weakly sclerotized, separated into anterior and posterior parts, anterior part with three shorter setae and one strongly sclerotized longer seta proximally, medial part with two groups of thinner setae arranged as short comb (ctenidium) on both sides, posterior half mostly membranous, central region with median wrench-like, slightly upcurved projection (Figs 6A, 7A and 7B), T11 weakly sclerotized, bearing short setae (for chaetotaxy see Fig 7A) with long, evenly upcurved median projection, with its apex above level of projection of T10, epiproct and paraproct unsclerotized; cerci (Figs 6D, 7A and 7B) unsegmented, longer than wide, conical with slightly pointed apex, covered with numerous minute spicules and one long distally oriented seta.

Abdominal sternite I (S1) scarcely sclerotized; sternites S2-S7 with sparse setation, distal edge of S8 narrow part with longer setae laterally, followed by weak depressions, each with one longer seta, middle part of distal edge (between depressions) concave; S9 narrow, trilobite distally, sparsely setose, S10 invisible externally, beneath S9, S11 with two lateral hemitergites, each with 2–3 setae of short and moderate length (Fig 7A).

Male genitalia symmetrical (Fig 7A–7C), composed of pair of dorsal lobate sclerites (sclerite Ia,b); mesal sclerite with trilobate posterior margin (sclerite II) and a pair of ventral sclerites (sclerite IIIa,b) with proximally rounded anterior processes and narrowed and posterior processes; inner parts of ventral sclerites with small sclerotized curved processes oriented antero-ventrally; weakly sclerotized (membranous) basal plate with tongue-like anterior process with rod-like paired sclerites medially (sclerites IVa,b). Anterior process of basal plate about 2 times longer than ventral sclerite. Intromittent organ long, encircling anterior process of basal plate (Fig 7B and 7C).

### Description of apterous female

Features of apterous female generally similar to those of apterous male except as follows: head without visible cephalic gland; metafemur slender, ventral surface with the same arrangement of spines, but spines thinner than in males; abdomen wider, maximal width 0.77–0.78 mm, abdominal T8 with only several longer setae, regularly rounded distally (Fig 6G); S8 strongly trapezoidal, wider than long, sparsely covered by short setae and several longer setae flanking distal and lateral edges; distal end with triangular projection medially (Fig 6H).

### Molecular barcode

We obtained partial 16S rRNA sequences (509 bp) of *L*. *gimmeli* sp. nov. as DNA barcode for the purpose of molecular identification of the species, and we deposited it in GenBank under accession number ON722348.

### Etymology

The species is named to honour its collector, Matthew L. Gimmel, from the Santa Barbara Museum of Natural History, USA. The gender is masculine.

### Distribution, site of collection

*Latinozoros gimmeli* sp. nov. was collected from under the bark of rotting *Pinus occidentalis* logs. The species is currently known from Dominican Republic, Trinidad, and Panama (see Discussion), but we expect its occurrence in similar habitats throughout Caribbean islands and Central America.

### Key to species *Latinozoros* species (based on males)

**Fig 8:**
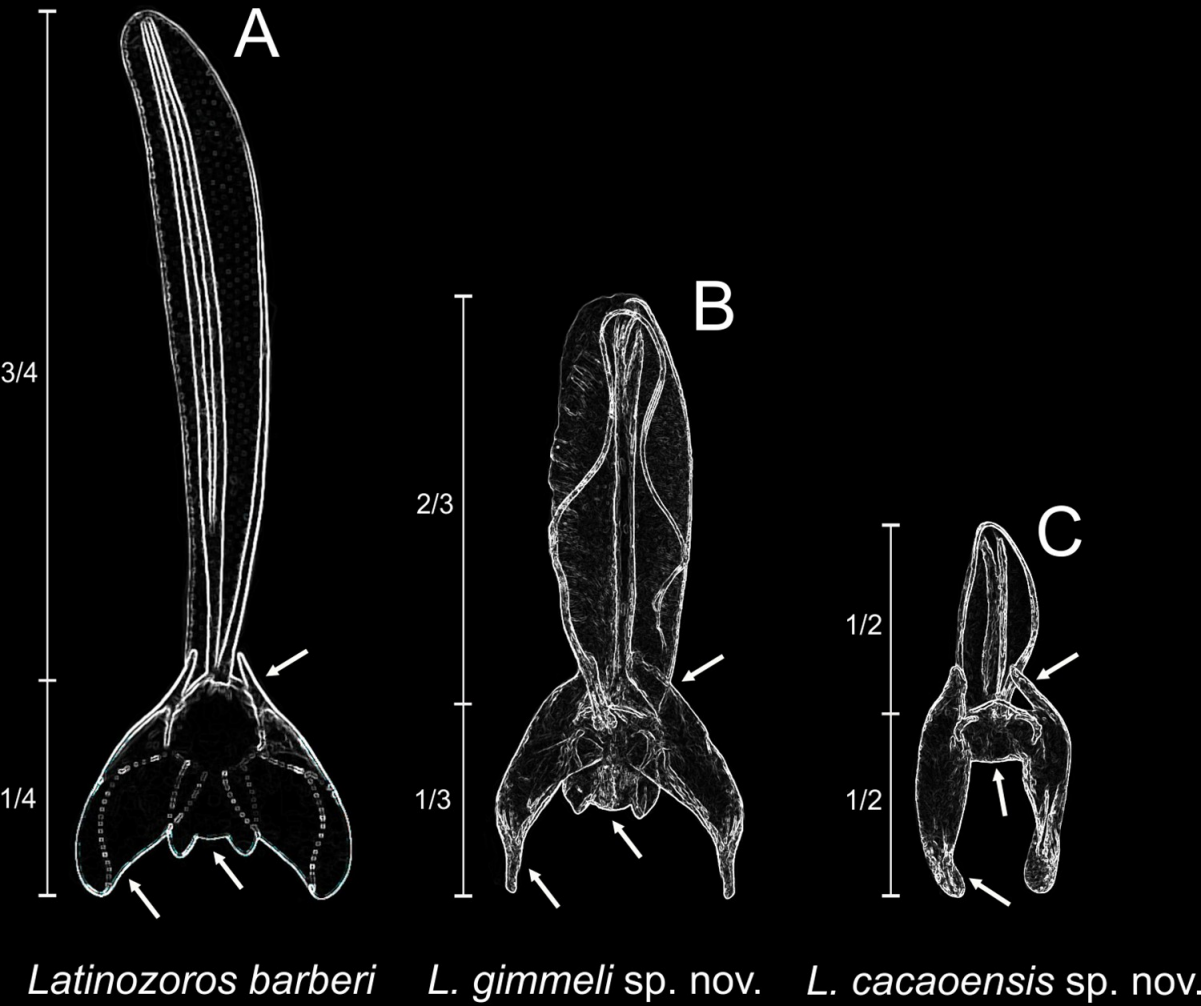
Characters of male genitals useful for the identification of *Latinozoros barberi* (Gurney, 1938) [30], *L*. *gimmeli* sp. nov., and *L*. *cacaoensis* sp. nov. The drawing of the male genital of *L*. *barberi* is adopted from Gurney (1938); the depicted male genitals of *L*. *gimmeli* sp. nov. and *L*. *cacaoensis* sp. nov. are original photographs that were improved by highlighting the edges using the filter “glowing outlines”in Photoshop CS6 Extended v13. Dorsal sclerites are masked for better visibility of important characters.

1. Anterior process of basal plate of male genital short (Fig 8C), as long as ventral sclerite (sclerite III). Distal processes of ventral sclerites long, apexes rounded. Ventral surface of metafemur with 7–8 stout long spurs. …. *L*. *cacaoensis* sp. nov.
  - Anterior process of basal plate of male genital long (Fig 8A and 8B), at least 2 times longer than ventral sclerite (sclerite III). Distal processes of ventral sclerites very short, with rounded apex, or long with distal apex narrowed and pointed. Ventral surface of metafemur with 9 stout long spurs. …. 2
2. Anterior process of basal plate of male genital long (Fig 8A), 3 times longer than ventral sclerite (sclerite III). Posterior processes of ventral sclerites robust, rounded (Fig 8A). Metafemur wider, length to maximum width ratio 2.1; dorsal surface of metafemur regularly rounded…. *L*. *barberi* (Gurney, 1938)
  - Anterior process of basal plate of male genital shorter (Fig 8B), about 2 times longer than ventral sclerite (sclerite III). Posterior processes of ventral sclerites long, narrowed. Metafemur narrower, length to maximum width ratio 2.5; dorsal surface of metafemur sigmoid…. *L*. *gimmeli* sp. nov.

### Phylogeny

**Fig 9:**
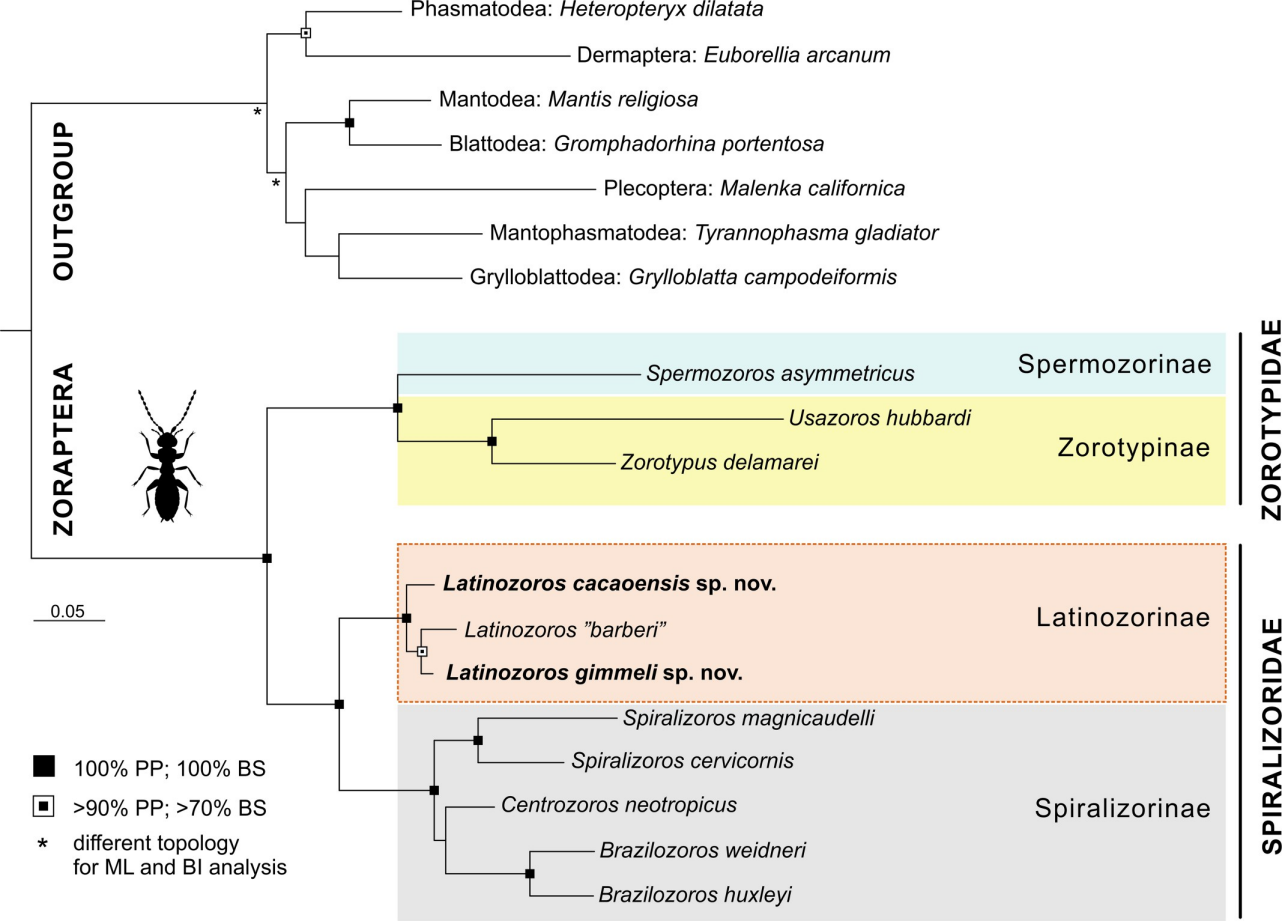
Phylogenetic tree of Zoraptera resolved by Bayesian inference based on the combined dataset for three molecular markers (18S, H3, and 16S). Bayesian posterior probabilities (PP) and RAxML bootstrap supports (BS) are expressed. For species in quotation marks, see Materials and Methods.

The aligned dataset of the three genes (18S, H3, and 16S) consisted of 3,073 bp, of which 753 bp were excluded by GBlock. According to a test for substitution saturation in DAMBE, none of the markers was saturated. PartitionFinder identified five partitions (18S, 16S, and three H3 codon positions) as the optimal partitioning scheme for phylogenetic analyses and selected the best-fit nucleotide substitution models for BI (S2 Table**)**. The phylogenetic trees based on the BI and ML methods and including representatives of seven genera from two known zorapteran families and four subfamilies were identical in topology and branch-support values. The families Zorotypidae and Spiralizoridae as well as the subfamilies Latinozorinae, Spiralizorinae, Spermozorinae, and Zorotypinae were strongly supported in both phylogenetic analyses (Fig 9). The three lineages of *Latinozoros*, two of which are described here as new species, were also well-supported.

Based on the Kimura 2-parameter model the minimum genetic divergence of 16S rRNA between *Latinozoros* species was 13% (*L*. *“barberi”* vs. *L*. *gimmeli* sp. nov.); the maximum genetic divergence was 21% (*L*. *“barberi”* vs. *L*. *cacaoensis* sp. nov.).

## Discussion

Current studies suggest that the order Zoraptera is distinctly more diverse than previously thought [3,6], and further research will likely increase the number of known species. The current study is focused on the genus *Latinozoros*, within which only one species *Z*. *barberi* had been previously recognized [5,35,36]. Although the occurrence of this species has been repeatedly reported from a number of localities in South and Central America, its status as a complex of several species had remained unrecognized until now. Our previous molecular phylogenetic study [3] revealed significant genetic differences between two studied populations, and the subsequent detailed morphological comparisons presented in this report led to the differentiation and description of two previously unknown species. All three known species are morphologically very similar, but are well-defined mainly based on characters of the male genitalia, but also based on features of distal abdominal sclerites and the legs.

The results of our phylogenetic analysis (Fig 9) confirmed the monophyly of the subfamily Latinozorinae and their sister position to Spiralizorinae, which is in line with previous studies by Kočárek et al. [3] and Matsumura et al. [6]. Together, both clades represent the family Spiralizoridae with a characteristic symmetrical male genitals, which Matsumura et al. [6] considered as the ancestral state in Zoraptera. The synapomorphy of Spiralizoridae (Latinozorinae+Spiralizorinae) is the male genital with developed long sclerotized intromittent organ, which encircling anterior membranous projection (apomorphy of Latinozorinae) or which is dorso-ventrally spirally coiled (apomorphy of Spiralizorinae). In contrast, the representatives of family Zorotypidae have asymmetrical male genitals with not developed intromittent organ [3].

**Fig 10:**
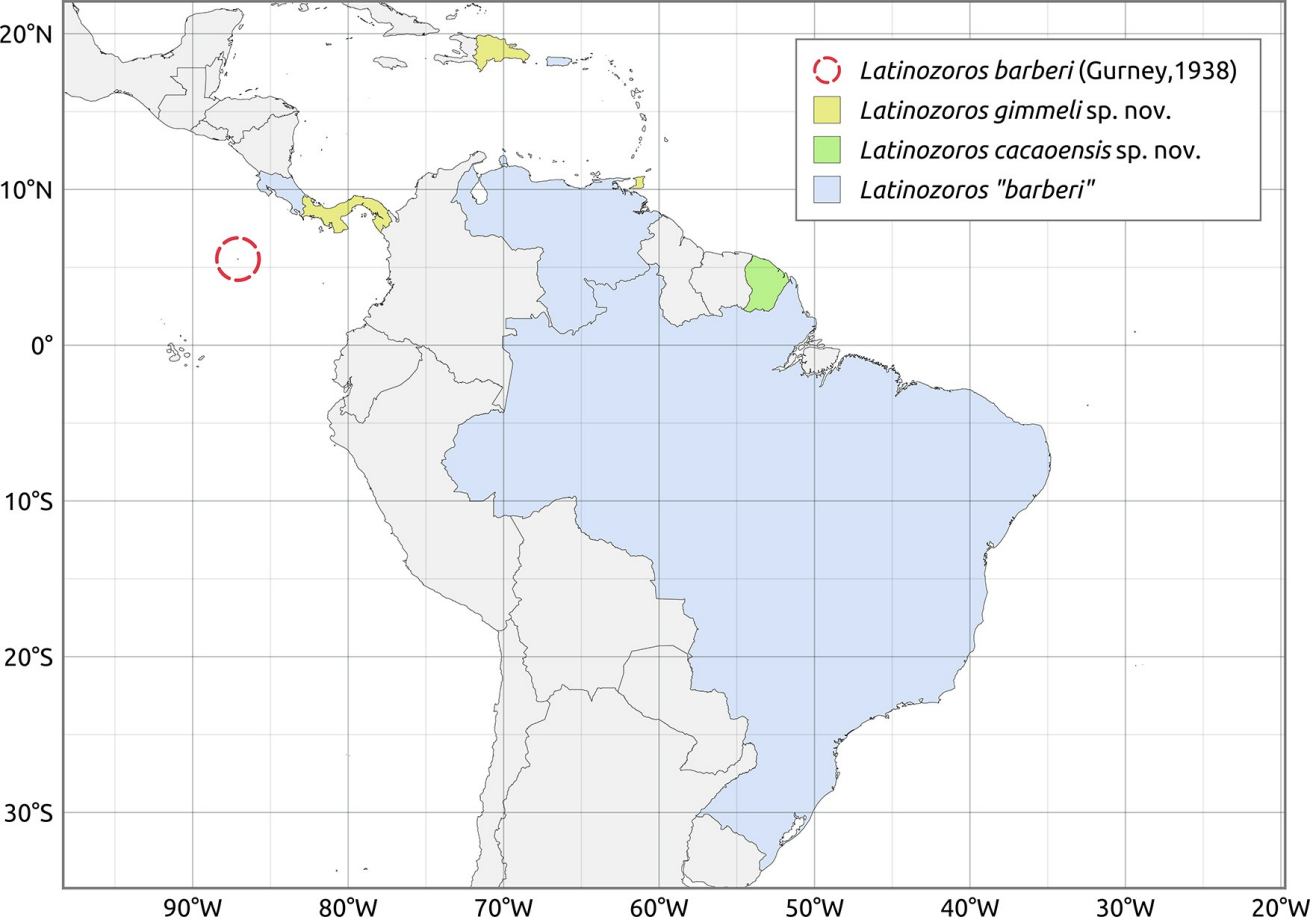
Known distribution of *Latinozoros* species. The map was created using *Natural Earth*, free vector and raster maps (https://www.naturalearthdata.com). For species in quotation marks, see Materials and Methods.

Although several previous studies provided direct or indirect information about the occurrence of *L*. *barberi*, as well as its biology and ecology, a number of them did not illustrate critical diagnostic characters in drawings, photographs, or detailed verbal descriptions [e.g., 6,28,32,33–35,37,38]. Based on these publications, it is therefore not possible to determine which species of the genus *Latinozoros* have or have not been studied, and future studies will be needed to review the occurrence of individual *Latinozoros* species and their areas of distribution (Fig 10). Choe [31] published a taxonomical redescription of *L*. *barberi* based on material collected in Panama, Costa Rica, and the Dominican Republic. Although Choe [31] illustrated important diagnostic characters, even that report fails to clarify areas of distribution of the *L*. *barberi* newly described here. The illustrations in Choe [31] show characters of both *L*. *gimmeli* (Figs 7–9 on page 151) and *L*. *cacaoensis* (Fig 6 on page 151), and the author simultaneously studied material from Panama, Costa Rica, and the Dominican Republic, but did not state which individuals were used to illustrate the relevant characters. However, ethological observations of *Latinozoros* from Panama [37] are accompanied by SEM photographs of tips of male and female abdomen, and visible diagnostic characters indicate that this population is *L*. *gimmeli* sp. nov. According to current knowledge, *L*. *gimmeli* sp. nov. likely occurs throughout the Caribbean islands and in Central America (at least in Panama). Because the majority of populations recorded as *L*. *barberi* have been published without information about the diagnostic characters, we have to assign to this species only the male collected on Cocos Island (Costa Rica) in the Atlantic [30]. Although *L*. *barberi* might be endemic to Cocos Island, the species might also occur on the Amazonia mainland. Currently known distribution of *Latinozoros* is summarized in Fig 10.

The biology of Zoraptera is poorly known [2,5]. Zorapterans are usually found in colonies under the bark of decaying logs [5] and seem to be primarily opportunistic omnivores feeding on fungal hyphae and spores, as well as on dead arthropods; they also may occasionally act as predators, capturing and eating small mites, collembolans, and nematodes [2]. Shetlar [39] identified remnant body parts of small arthropods in the guts of freshly killed *Usazoros hubbardi* (Caudell, 1918), and Choe [32] observed cannibalistic behaviour in *L*. *barberi* and *Centrozoros gurneyi* (Choe, 1989) [31] under laboratory conditions. Our observations of the visible contents of the gastrointestinal tract of a male *L*. *cacaoensis* sp. nov. revealed a prevalence of fungal hyphae and spores in the natural diet, but additional research is needed to evaluate the feeding habit of this and other species.

The descriptions of *L*. *cacaoensis* sp. nov. and *L*. *gimmeli* sp. nov. provided in the current study increase the number of described Zoraptera species to 60; this number includes 45 extant species and 15 fossil species known from Cretaceous (12) and Miocene (3) amber [40,41]. Zorapterans are inconspicuous in appearance and live secretly, but they appear to occur in most tropical areas worldwide, with no species yet reported from some large and potentially suitable areas (e.g., Myanmar, Thailand, Cambodia, Sulawesi, Moluccas or Papua in South-Eastern Asia, or tropical countries in equatorial Africa). The species diversity is likely to be much higher than previously recognized, and a significant increase in the number of known species can be expected in the future.

## Supporting information

S1 Table Primers and PCR conditions used for amplification. (PDF)

S2 Table Alignment length and best-fit substitution models determined by PartitionFinder. Models for protein-coding gene, histone 3, are shown for the 1^st^, 2^nd^ and 3^rd^ codon positions.
(PDF)
