# Supplementary material for: Cryptic diversity in Zoraptera: Latinozoros barberi (Gurney, 1938) is a complex of at least three species (Zoraptera: Spiralizoridae)
Source: PLoS One. 2023 Jan 25;18(1):e0280113. doi: 10.1371/journal.pone.0280113 (PMC9876274; doi:10.1371/journal.pone.0280113)
Supplement: S1 Table — (PDF) [file pone.0280113.s001.pdf]

S1 Table. Primers and conditions used for PCR amplification.

Gene	Primer name	Primer sequence 5'→3'	PCR protocol (this study)	Primer reference
**16S**	ar	CGCCTGTTTATCAAAAACAT	3 min at 93 °C, 35x (30 s at 93 °C, 1 min at 48 °C, 90 s at 72 °C), 5 min at 72 °C	Palumbi et al. 2002
	br	CCGGTCTGAACTCAGATCACGT		
**18S**	F	AACCTGGTTGATCCTGCCAGT	3 min at 95 °C, 35x (30 s at 95 °C, 40 s at 51 °C, 2 min at 72 °C), 10 min at 72 °C	Katana et al., 2001
	R	TGATCCTTCTGCAGGTTCACCTACG		
**H3**	AF	ATGGCTCGTACCAAGCAGACVGC	3 min at 94 °C, 33x (30 s at 94 °C, 30 s at 49 °C, 50 s at 72 °C), 3 min at 72 °C	Colgan et al., 2008
	AR	ATATCCTTRGGCATRATRGTGAC		

References

Colgan, D.J., Hutchings, P.A., Beacham, E., 2008. Multi-gene analyses of the phylogenetic relationships among the Mollusca, Annelida, and Arthropoda. Zoological Studies 47, 338-351.

Katana, A., Kwiatowski, J., Spalik, K., Zakryś, B., Szalacha, E., Szymańska, H., 2001. Phylogenetic position of Koliella (Chlorophyta) as inferred from nuclear and chloroplast small subunit rDNA. Journal of phycology, 37(3), 443-451.

Palumbi, S.; Martin, A.; Romaro, S.; McMillan, W.O.; Stice, L.; Grabowski, G., 2002. The simple fool's guide to PCR. Version 2.0, October 29, University of Hawaii, Honolulu, 1-45.
