# Supplementary material for: Cryptic diversity in Zoraptera: Latinozoros barberi (Gurney, 1938) is a complex of at least three species (Zoraptera: Spiralizoridae)
Source: PLoS One. 2023 Jan 25;18(1):e0280113. doi: 10.1371/journal.pone.0280113 (PMC9876274; doi:10.1371/journal.pone.0280113)
Supplement: S2 Table — Models for protein-coding gene, histone 3, are shown for the 1st, 2nd and 3rd codon positions. (PDF) [file pone.0280113.s002.pdf]

S2 Table. Best-fit substitution models determined by PartitionFinder, and alignment lengths. Models for protein-coding gene, histone 3 (H3), are shown for the 1st, 2nd and 3rd codon positions.

**Gene**	**Length (bp)**	**GBlock length (bp)**	**Substitution models (AICc criterion)**
H3	341	341	GTR+I, JC, GTR+G
16S	554	407	GTR+I+G
18S	2178	1572	GTR+I+G
**Total**	**3073**	**2320**	
